# Seroprevalence of *Toxoplasma gondii* and Feline Immunodeficiency Virus in Domestic Cats and Their Associations with Clinical Signs

**DOI:** 10.1007/s11686-026-01334-w

**Published:** 2026-07-04

**Authors:** Eva Bártová, Kamil Sedlák, Špela Vodlan, Marie Budíková, Karol Račka

**Affiliations:** 1 Faculty of Veterinary Hygiene and Ecology, Department of Biology and Wildlife Department, University of Veterinary Sciences Brno, Brno, Czechia; 2 State Veterinary Institute Prague, Prague, Czechia; 3 Animalia, Veterinarska ambulanta Zagorje, Zagorje ob Savi, Slovenia; 4 Faculty of Science, Department of Mathematics and Statistics, Masaryk University, Brno, Czechia; 5 Department of Epizootology, Parasitology and Protection of One Health, University of Veterinary Medicine and Pharmacy in Košice, Košice, Slovakia

**Keywords:** *Felis catus*, Toxoplasmosis, Retrovirus, Co-infection, ELISA, Seropositivity

## Abstract

**Purpose:**

Feline immunodeficiency virus (FIV) induces immunosuppression and may predispose cats to opportunistic infections, including *Toxoplasma gondii*. Data on natural coinfections in domestic cats remain limited, particularly in Europe.

**Methods:**

A total of 105 domestic cats from veterinary clinics and shelters in Slovenia and the Czech Republic were examined. Antibodies to *T. gondii* were detected by Enzyme-Linked Immunosorbent Assay, and FIV antibodies by an immunochromatographic test. Associations with sex, age, housing conditions, and clinical signs were analysed using appropriate statistical tests.

**Results:**

Antibodies to *T. gondii* were detected in 18.1% of cats, FIV antibodies in 10.5%, and coinfection in 4.8%. *T. gondii* seropositivity was significantly associated with young age, pet ownership, and the presence of clinical signs. FIV seropositivity was more frequent in males, young cats, pet cats, and clinically affected animals. Coinfection was observed more often in males and pet cats. Cats positive for *T. gondii* and/or FIV exhibited clinical signs significantly more frequently than seronegative cats (68% vs. 35%, *p* = 0.0036). Coinfected cats tended to present multiple categories of clinical signs more often than monoinfected cats, although this difference was not statistically significant.

**Conclusions:**

This study provides evidence of associations between host factors, *T. gondii* and FIV seropositivity, and clinical manifestations in naturally infected cats. Despite limitations related to sample size and serological testing, the findings contribute novel data on *T. gondii*/FIV coinfection in domestic cats in Central Europe.

## Introduction

Clinical toxoplasmosis is relatively rare in cats. Nevertheless, cats and other felids, as the definitive hosts of *Toxoplasma gondii*, can shed oocysts in their faeces and thereby contaminate the environment. Cats living in close contact with humans may also contribute to the transmission of *T. gondii* infection, although oocysts shedding by adult immunocompetent cats is generally uncommon. In immunosuppressed felids, recurrent shedding appears to occur more frequently [13]. Furthemore, *T. gondii* infection may result in severe clinical disease and can even be fatal in immunocompromised cats [1]. The prevalence of *T. gondii* infection in cats varies, depending on their feeding habits and appears to be generally higher in feral cats that hunt wild rodents and birds, compared to indoor pet cats that do not have contact with wild animals [5]. Apart from *T. gondii*, cats are sensitive to other infectious pathogens, mainly to those of viral origin, such as Feline Immunodeficiency Virus (FIV) and Feline Leukaemia Virus (FeLV), belonging to retroviruses. Feline retroviral infections can lead to clinical diseases or to immunosuppression that predispose cats to other infections [1, 5]. Thus, FIV or FeLV infections can predispose cats to acute toxoplasmosis, often with severe respiratory signs [3, 15]. Few studies have investigated *T. gondii* and FIV coinfections. Although the interaction between *T. gondii* and FIV has been investigated experimentally and in a limited number of field studies [3, 6, 9, 10, 12, 18], data on naturally occurring coinfections in domestic cats remain relatively scarce, particularly in Europe. Therefore, this study aimed to detect antibodies to both pathogens in naturally infected domestic cats and assess whether coinfection is associated with clinical signs.

## Materials and Methods

Blood samples used in this study were obtained by veterinarians by jugular venipuncture from 105 domestic cats (*Felis catus*) in shelters (*n* = 90) or veterinary clinics (*n* = 15) in Slovenia (*n* = 84) and the Czech Republic (*n* = 21). The Slovenian cats originated from the central part of the country (Ljubljana region), whereas the Czech cats originated from the South Moravian Region. Cats were enrolled on an opportunistic basis during routine veterinary examinations in clinics or health monitoring in shelters. No animals were selected based on suspected toxoplasmosis, FIV infection, or specific clinical signs. Before blood sampling, the clinical examination of cats was done by veterinarians. The cats included females (*n* = 55) and males (*n* = 50), aged from 1 month to 14 years. For this study, cats were divided into 2 age categories: young 0–1 years (*n* = 39) and adults 1 year (*n* = 66). The cats were either without evident clinical signs (*n* = 60), or were suffering from some of the signs or their combinations (*n* = 45) e.g. neurological abnormalities, upper and lower respiratory tract infections, ocular disorders (conjunctivitis), gastrointestinal disorders, renal failure, skin lesions (dermatitis/dermatosis), generalised lymphadenopathy, stomatitis/gingivitis, and nonspecific signs (anorexia and weight loss). The evaluated clinical signs were selected because they have previously been associated with toxoplasmosis, FIV infection, or both diseases. Neurological, ocular, respiratory and nonspecific systemic signs have been reported in feline toxoplasmosis, whereas generalized lymphadenopathy, stomatitis/gingivitis, dermatological lesions and chronic respiratory disorders are commonly described in FIV-infected cats. Blood samples were centrifuged at 1500 × g for 10 min within 24 h after collection. The separated sera were stored at − 20 °C until serological examination.

For detection of antibodies to *T. gondii*, a commercial Enzyme-Linked Immunosorbent Assay (ID Screen^®^ T. gondii Indirect Multi-species, IDvet, Grabels, France) was used according to the manufacturer’s instructions. The manufacturer reports validation of the assay for multiple animal species, including cats; however, species-specific diagnostic sensitivity and specificity values for cats are not provided in the publicly available technical documentation. Samples with S/*P* ≥ 50% were considered positive. Antibodies to FIV were detected by immunochromatographic screening test (Rapid FIV Ab/FeLV Ag Kit, BIONOTE, Hwaesong, Korea), according to the manufacturers’ instructions. According to the manufacturer, the FIV antibody assay has a diagnostic sensitivity of 96.8% and specificity of 99.6% when compared with Western blot.

The results were statistically analysed, considering the variables of sex (female, male), age category (kittens < 1 year, adult ≥ 1 year), way of keeping (pet cats from veterinary clinic, stray cats from shelters), and the presence of clinical signs (yes, no). The data analysis was performed with the Pearson Chi-Square test of independence, Monte Carlo test or Fisher’s exact test, using STATISTICA 14 [17] or IBM SPSS Statistics 30.0 [7]. We tested the null hypothesis that an occurrence of antibodies to individual pathogens (*T. gondii* or FIV) and the occurrence of clinical signs do not depend on the given variables (sex, age category, way of keeping, and clinical signs). The differences were considered statistically significant when the *p*-value was ≤ 0.05.

## Results

Antibodies to *T. gondii* were detected in 19 cats (18.1%) and antibodies to FIV in 11 cats (10.5%), with monoinfection (*T. gondii* or FIV) in 20 cats (19.0%) and coinfection in 5 cats (4.8%). Among *T. gondii*-seropositive cats, S/P values ranged from 51% to 146% (median 99%) in cats with *T. gondii* monoinfection and from 74% to 153% (median 125%) in coinfected cats. Due to the small number of coinfected animals, no statistical comparison of S/P values between groups was performed. The seroprevalence of *T. gondii* infection was 20% in females and 16% in males; however, this difference was not statistically significant (*p* = 0.5949). *T. gondii* antibodies were significantly more frequent in young than in adult cats (33% vs. 9.1%, *p* = 0.0018), in pet compared to stray cats (60% vs. 11.1%, *p* = 0.0001), and in cats with clinical signs compared to those without (26.7% vs. 11.7%, *p* = 0.0482).

FIV antibodies were detected more often in males than in females (20% vs. 1.8%, *p* = 0.0024), in pet compared to stray cats (26.7% vs. 7.8%, *p* = 0.0494), and in cats with clinical signs compared to those without (20% vs. 3.3%, *p* = 0.0085). FIV antibodies were also detected significantly more often in young cats compared to adults (25.6% vs. 1.5%, *p* = 0.0002). However, a detailed analysis of the age distribution within the young category (0–1 year) revealed that 70% (7/10) of the seropositive individuals were kittens aged 3–4 months. All these kittens originated from a single location (Slovenian shelter), suggesting a high probability of maternal antibody interference.

Coinfection of *T. gondii* and FIV was observed more frequently in males than in females (10% vs. 0%, *p* = 0.0219) and in pet compared to stray cats (20% vs. 2.2%, *p* = 0.0202). In young cats, the prevalence of coinfection was 10.3% compared to 1.5% in adults, suggesting a trend toward higher prevalence in young cats, but the difference was not statistically significant (*p* = 0.0622). Similarly, coinfection was observed in 8.9% of cats with clinical signs compared to 1.7% of cats without, but this difference was not significant (*p* = 0.1618). The results are summarised in Table 1. In total, 45 of 105 cats (42.9%) showed some clinical signs; in 17 of them (37.8%), antibodies to *T. gondii* and/or FIV were detected.

**Table 1:** Results of serological examination of domestic cats for antibodies to *Toxoplasma gondii* and feline immunodeficiency virus (FIV) according to sex, age, way of keeping, and occurrence of clinical signs

Characteristic	Total	Monoinfection (*T. gondii* or FIV)	*T. gondii*	*p*-value	FIV	*p*-value	Co-infection *T. gondii* and FIV	*p*-value
Sex				0.5949		**0.0024**		**0.0219**
Female	55	12 (21.8%)	11 (20%)		1 (1.8%)		0 (0%)	
Male	50	8 (16%)	8 (16%)		10 (20%)		5 (10%)	
Age				**0.0018**		**0.0002**		0.0622
Young (≤ 1 year)*	39	15 (38.5%)	13 (33%)		10 (25.6%)		4 (10.3%)	
Adult (> 1 year)	66	5 (7.6%)	6 (9.1%)		1 (1.5%)		1 (1.5%)	
Way of keeping				**0.0001**		**0.0494**		**0.0202**
Pet cats (from clinics)	15	7 (46.7%)	9 (60%)		4 (26.7%)		3 (20%)	
Stray cats (from shelters)	90	13 (14.4%)	10 (11.1%)		7 (7.8%)		2 (2.2%)	
Clinical signs				**0.0482**		**0.0085**		0.1618
Yes	45	13 (28.9%)	12 (26.7%)		9 (20%)		4 (8.9%)	
No	60	7 (11.7%)	7 (11.7%)		2 (3.3%)		1 (1.7%)	
Total	105	20 (19.0%)	19 (18.1%)		11 (10.5%)		5 (4.8%)	

Data are presented as numbers and percentages. Depending on the expected frequencies, either Pearson’s chi-square test or Fisher’s exact test was used; *p*-values ≤ 0.05 were considered statistically significant and are marked with an asterisk

*Young category included 7 kittens < 6 months of age with possible maternal antibodies

**Fig. 1:**
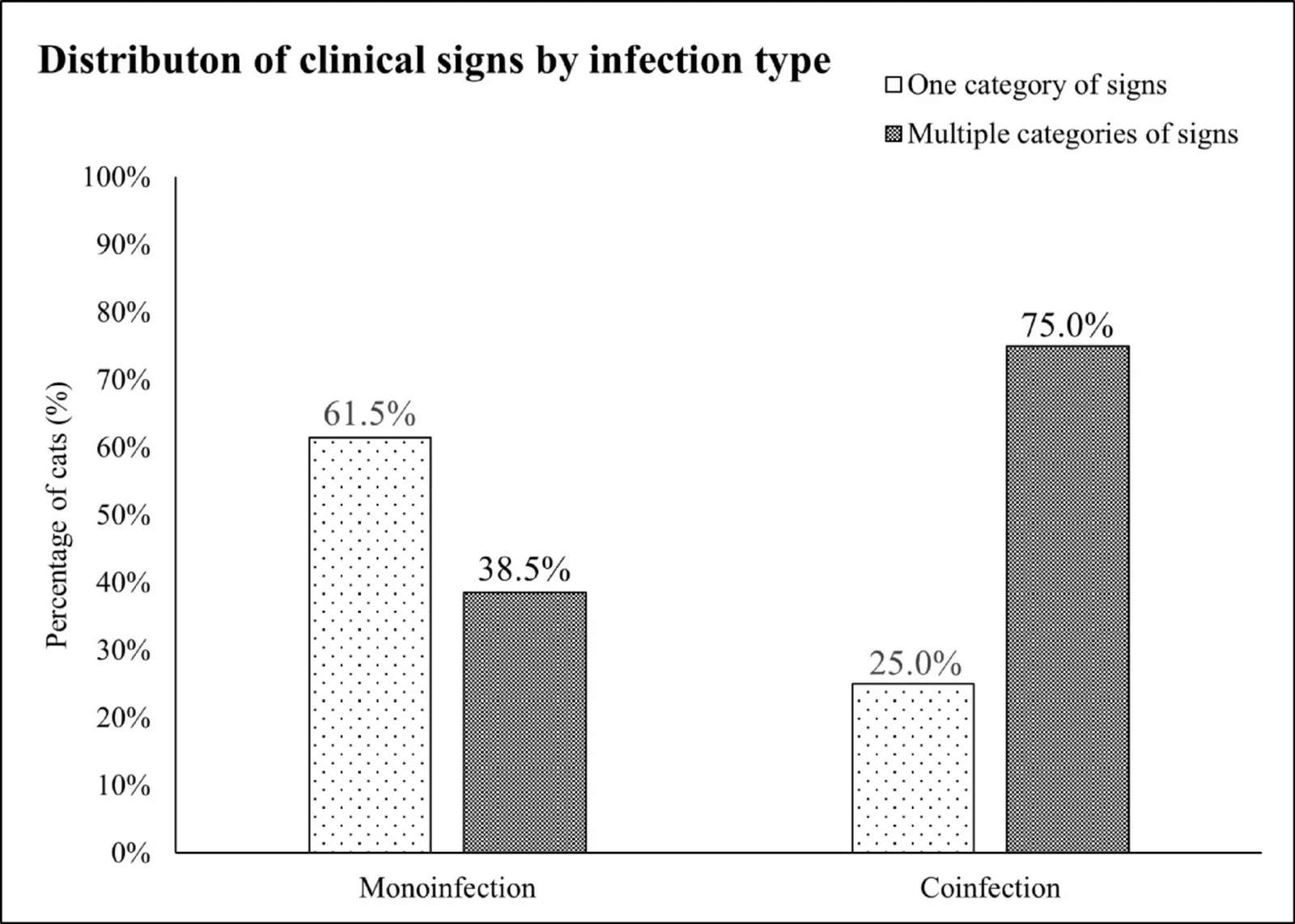
Distribution of clinical signs in cats with monoinfection (*Toxoplasma gondii* or FIV) and coinfection (*Toxoplasma gondii* and FIV). Bars show the percentage of cats with only one category of clinical signs (light bars) and with multiple categories of clinical signs (dark bars). Coinfected cats more frequently exhibited multiple categories of signs compared with monoinfected cats, although the difference was not statistically significant

Cats, positive for *T. gondii* and/or FIV exhibited clinical signs more often than cats without these infections (68% vs. 35%, *p* = 0.0036). Respiratory disorders were among the most common clinical findings in both monoinfected and coinfected cats. Coinfected animals additionally showed combinations of ocular, neurological, musculoskeletal, and lymphatic abnormalities. In contrast, monoinfected cats most often exhibited only a single category of clinical signs, whereas coinfected cats tended to present multiple concurrent clinical manifestations affecting several organ systems. Clinical signs were observed in 80% of coinfected cats (4/5) compared with 65% of cats with monoinfection (13/20), but this difference was not statistically significant (OR = 2.15, *p* = 0.5268). Cats with coinfection more frequently exhibited multiple categories of clinical signs (3/4 coinfected cats, 75%) compared with monoinfected cats (5/13, 38%). In contrast, monoinfected cats more often showed only a single clinical sign (62% vs. 25%). This pattern is illustrated in Fig. 1; Table 2, which shows the distribution of clinical manifestations by infection type.

**Table 2:** Categories of clinical signs in domestic cats with monoinfection (*Toxoplasma gondii* or FIV) or coinfection (*Toxoplasma gondii* and FIV)

Category of clinical signs	Monoinfection (*T. gondii* or FIV)	*T. gondii*	FIV	Co-infection (*T. gondii* and FIV)
One category	8	6	2	1
Neurological abnormalities	1	1		
Upper respiratory tract infection	1	1		
Ocular disorders	1	1		
Oral cavity infection	2	1	1	
Gastrointestinal disorder	1	1		
Renal failure	1		1	
Nonspecific signs	1	1		1
More categories	5	2	3	3
Lower and upper respiratory tract infection	2	1	1	
Lower and upper respiratory tract infection, ocular disorders	1	1		
Upper respiratory tract infection, skin lesions and nonspecific signs	1		1	
Ocular disorders, skin lesions and nonspecific signs	1		1	
Lower and upper respiratory tract infection, nonspecific signs				1
Lower and upper respiratory tract infection, ocular disorders, musculoskeletal system infection and nonspecific signs				1
Lower and upper respiratory tract infection, ocular disorders, neurological abnormalities, generalized lymphadenopathy and nonspecific signs				1
Total	13	8	5	4

The table shows whether cats exhibited only one category of signs or multiple categories of signs

## Discussion

Stray cats play an important role in the contamination of the environment with *T. gondii* oocysts that can be a source of infection for humans and for cats with outdoor access. Since cats with antibodies to *T. gondii* have already shed oocysts, the seropositivity thus indicates the proportion of cats that have a role in environmental contamination [15]. In our study, seropositivity to *T. gondii*, FIV, and their coinfection was significantly higher in pet cats than in stray cats (*p* ≤ 0.05). This finding contrasts with the results of many previous studies, in which higher seroprevalence was often reported in free-roaming or feral cats. However, detailed information regarding the lifestyle of pet cats included in the present study, such as outdoor access, hunting behaviour, and diet, was not available. Therefore, the observed differences should be interpreted with caution, as some pet cats may have had regular outdoor access and consequently a higher risk of exposure to infectious agents. In addition, shelter populations may include animals with diverse previous living conditions and durations of stay. Further studies including detailed lifestyle data would be necessary to clarify these associations.

It is possible that most of the infected stray cats die without any records. Since stray cats can be an important source of various infections [5, 8, 15], it is important to support preventive programs in shelters, including castration, vaccination, quarantine, diagnosis and the treatment of infectious diseases. There are recent studies confirming higher *T. gondii* seroprevalence in naturally infected cats with simultaneous FIV infection. Previous studies have reported inconsistent results regarding the association between *T. gondii* and FIV infections. In a study from Brazil, Feitosa et al. [6] reported a higher frequency of *T. gondii* seropositivity among FIV-positive cats, with coinfection detected in 75% of cats compared with 53.4% of cats seropositive only for *T. gondii* and 23.3% seropositive only for FIV. In contrast, Bezerra et al. [2] found a lower frequency of *T. gondii*/FIV coinfection (12.5%) than either *T. gondii* infection alone (25.8%) or FIV infection alone (35%) in another Brazilian cat population. These discrepancies may reflect differences in study design, geographical location, environmental exposure, and characteristics of the sampled cat populations. While several studies from South America have explored these associations, there is a significant lack of data from the European continent. To date, only a study conducted in Belgium [4] has demonstrated an association between FIV and *T. gondii* seropositivity. Our study, however, provides the first evidence from Central Europe linking this coinfection directly to the complexity of clinical manifestations. Unlike previous European surveys that focused primarily on prevalence, our data show that coinfected cats in this region are significantly more likely to exhibit multiple categories of clinical signs, highlighting a synergy between these pathogens that has been underreported in European feline populations.

In our study, cats with coinfection of *T. gondii*/FIV also had higher antibodies’ S/P values (74−153%, median 125%) than cats with monoinfection of *T. gondii* (51−146%, median 99%). However, because of the small number of coinfected animals, no statistical comparison was performed and no conclusions regarding the relationship between antibody levels and clinical severity can be drawn from the present dataset. However, in another study, cats with coinfection of FeLV/FIV had lower titres of *T. gondii* IgG antibodies than in cats that were negative for these retroviral infections [16]. Cats with coinfection of *T. gondii/*FIV can develop detectable IgG antibodies later than *T. gondii* positive/FIV negative cats [9, 12], or there can even be an absence of IgG *T. gondii* antibodies within two weeks after infection in cats with coinfection of FIV [3]. It is evident that there can be different results when testing antibodies to different pathogens, depending on the immunity of cats, the mutual effect of pathogens influencing the formation of antibodies, and the method used for screening. This is why caution should be taken when interpreting low titers of *T. gondii* antibodies in cats suspected of being infected with FIV [12].

Morbidity in *T. gondii* or FIV infected cats, was relatively high. However, clinical signs were more serious in cats with coinfection of *T. gondii*/FIV, similarly to experimental surveys [3, 12, 14]. Thus, we observed the effect of the mutual influence of *T. gondii*/FIV infections on the manifestation of clinical signs, because cats with monoinfection usually showed single clinical signs, while cats with coinfection more often displayed multiple clinical signs, including lower and upper respiratory tract infection, generalised lymphadenopathy, ocular disorders, neurological abnormalities, musculoskeletal system infection, and nonspecific signs. This finding is in accordance with the clinical signs of acute toxoplasmosis, which could be various, with fatal pneumonia being seen most often [5]. In addition, primary life-threatening pneumonia was also described as a main reason of morbidity in cats experimentally infected with *T. gondii* with FIV coinfection [3]. Based on experiments, there is a variability of clinical signs in cats, depending on the use of different *T. gondii* and FIV isolates, different routes of administration and whether *T. gondii* infection occurs before or after infection with FIV [3].

An unexpected finding was the higher *T. gondii* seropositivity observed in young cats compared with adults. Most epidemiological studies report increasing *T. gondii* seroprevalence with age as a consequence of cumulative exposure. The present result may reflect the relatively small sample size, local epidemiological conditions, or clustering of infections within specific groups of animals. Because serological testing detects previous exposure rather than active infection, temporal relationships between age and infection risk cannot be established. Therefore, this finding should be interpreted with caution and requires confirmation in larger studies. Regarding host-related factors, age is generally considered a significant predictor for FIV infection, with prevalence typically increasing as cats age due to cumulative exposure. However, our results showed a contrasting trend, with a significantly higher FIV seroprevalence at the young age group (25.6%) compared to adults (1.5%, *p* = 0.0002). A detailed retrospective review of our records revealed that 70% (7/10) of these young seropositive cats were kittens aged only 3–4 months, all originating from a single shelter environment. In this specific age group, clinical antibody-based assays (ELISA) often detect maternal antibodies (colostral immunity) rather than active infection; these antibodies can persist for up to 6 months [10]. This likely explains the disproportionately high seropositivity in our young group and reflects the infection status of the cat mothers or the high environmental pressure in the shelter rather than horizontal transmission among kittens. The absence of confirmatory PCR testing to distinguish between persistent infection and maternal immunity is a limitation of this finding, and these specific results should be interpreted as an indicator of early life exposure. Although, in some studies, there was no potentiating effect of *T. gondii* infection on the rate of FIV-induced immunosuppression [9], and no association between FIV infection and health status [15], the *T. gondii*/FIV challenge model was used to test the hypothesis that immune dysfunction occurs relatively early after FIV infection and that it may predispose cats to clinical disease [3]. In the case of naturally infected cats, it is more complicated, because we do not know the exact phase of FIV immunosuppression and whether *T. gondii* infection is a primary or reactivated latent infection. Although the cross-sectional design of the present study does not allow conclusions regarding causality or disease progression, our findings are consistent with experimental studies suggesting that interactions between FIV infection and *T. gondii* may influence the clinical expression of both infections. It seems that FIV infection favours *T. gondii* proliferation and *T. gondii* may increase immunosuppression, probably by the production of the tumour necrosis factor. Therefore, the main result of this is a higher *T. gondii* replication and a rapid progression of disease [11, 12]. No clinical signs in 2 FIV-infected cats could be explained by the fact that the stage of retroviral infection and related immunosuppression are important factors for the development of clinical diseases and also for potential treatment.

The present study demonstrates significant associations between *Toxoplasma gondii* and FIV seropositivity, host-related factors, and clinical manifestations in domestic cats. Although limited by sample size and the use of serological methods, the findings highlight the potential clinical relevance of *T. gondii*/FIV coinfection and provide novel epidemiological data from Central Europe. Further large-scale studies are warranted to clarify the temporal and causal relationships between these infections.

## Data Availability

No datasets were generated or analysed during the current study.
